# Study on Lesion Assessment of Cerebello-Thalamo-Cortical Network in Wilson's Disease with Diffusion Tensor Imaging

**DOI:** 10.1155/2017/7323121

**Published:** 2017-07-11

**Authors:** Anqin Wang, Hongli Wu, Chunsheng Xu, Lanfeng Tang, Jaeyoun Lee, Min Wang, Man Jiang, Chuanfu Li, Qi Lu, Chunyun Zhang

**Affiliations:** ^1^The First Affiliated Hospital of Anhui University of Chinese Medicine, Hefei, Anhui 230012, China; ^2^Laboratory of Digital Medical Imaging, Medical Imaging Center, Hefei, Anhui 230012, China; ^3^Medical Information Engineering, Anhui University of Chinese Medicine, Hefei, Anhui 230012, China

## Abstract

Wilson's disease (WD) is a genetic disorder of copper metabolism with pathological copper accumulation in the brain and any other tissues. This article aimed to assess lesions in cerebello-thalamo-cortical network with an advanced technique of diffusion tensor imaging (DTI) in WD. 35 WD patients and 30 age- and sex-matched healthy volunteers were recruited to accept diffusion-weighted images with 15 gradient vectors and conventional magnetic resonance imaging (MRI). The DTI parameters, including fractional anisotropy (FA) and mean diffusion (MD), were calculated by diffusion kurtosis estimator software. After registration, patient groups with FA mappings and MD mappings and normal groups were compared with 3dttest and receiver-operating characteristic (ROC) curve analysis, corrected with FDR simulations (*p* = 0.001, *α* = 0.05, cluster size = 326). We found that the degree of FA increased in the bilateral head of the caudate nucleus (HCN), lenticular nucleus (LN), ventral thalamus, substantia nigra (SN), red nucleus (RN), right dentate nucleus (DN), and decreased in the mediodorsal thalamus and extensive white matter. The value of MD increased in HCN, LN, SN, RN, and extensive white matter. The technique of DTI provides higher sensitivity and specificity than conventional MRI to detect Wilson's disease. Besides, lesions in the basal ganglia, thalamus, and cerebellum might disconnect the basal ganglia-thalamo-cortical circuits or dentato-rubro-thalamic (DRT) track and disrupt cerebello-thalamo-cortical network finally, which may cause clinical extrapyramidal symptoms.

## 1. Introduction

Progressive hepatolenticular degeneration, or Wilson's disease (WD), is a genetic disorder of copper metabolism [1]. Alterations in the gene, which is responsible for the synthesis of protein P-type ATPase (ATP7B), lead to pathological copper accumulation, especially in the liver and the brain [2]. Copper accumulation usually causes severe clinical manifestations, including liver failure, hepatitis [3], cirrhosis [4], premature death [5], and motor [6], emotion, cognitive impairments [7]. The neuropathological hallmark of the disease is neuronal loss and atrophy in lenticular nucleus, as well as in the midbrain [8], thalamus, and other parts of the basal ganglia [5].

Conventional magnetic resonance imaging (MRI) is the popular radiological technique for diagnosis and follow-up of WD [9–11]. Areas of symmetric hypointensity on T1-weighted images and symmetric hyperintensity on T2-weighted images were presented in the basal ganglia, thalamus, and brain stem [12]. Diffusion tenser imaging (DTI) is a noninvasive technique to measure microstructural organization in brain tissues in vivo, based on the properties of water diffusion [13]. In previous studies, DTI has been applied to assess the microstructure of thalamus and evaluate diffusion abnormalities in the white matter regions in WD patients [14, 15]. However, the basal ganglia, which is the most commonly damaged in Wilson's disease, was not detected. And these region-specific approaches assume consistency of effects amongst all WD patients, which may not be a valid assumption. The current study aim to evaluate microstructure of the whole brain with DTI technique and the probably correlation between the areas of abnormalities and clinical symptoms.

## 2. Materials and Methods

### 2.1. Subjects

This study was approved by the human research committee in the First Affiliated Hospital of Anhui University of Chinese Medicine (AUCM), and all the patients signed informed consent for their participation in the study. In this project, a total of 35 WD patients and 30 age- and sex-matched healthy volunteers were recruited. The patients were enrolled from the Department of Neurology of the First Affiliated Hospital of AUCM (21 males and 14 females), ranging in age from 18 to 45 years with mean age of 26.94 ± 6.98 years. The diagnosis of WD based on the clinical manifestations (extrapyramidal symptom, pyramidal symptom, and behavioral problems), presence of the Kayser-Fleischer (KF) ring, low serum total copper and ceruloplasmin level, decreased activity of copper-dependent oxidase, and increased 24 h urinary excretion of copper. In this study, clinical features of patients with WD were as follows: extrapyramidal symptom (100%), KF ring (100%), copper-dependent oxidase activity (0.04 ± 0.06/L), and 24 h urinary excretion of copper (873.3 ± 538.8 *μ*mmol/L), mean duration of illness (93.9 ± 60.5 months), and family history (23%) with the modified Goldstein classification [16] ranging from II to IV. None of the patients had a history of neurological diseases other than WD. For controls, healthy subjects (15 males and 15 females) were enrolled from college students or society officers without central nervous system diseases, mental diseases, and other serious diseases, ranging in age from 19 to 48 years with mean age of 26.2 ± 5.62 years. The details are mentioned in Table 1.

### 2.2. MRI Protocol

All participants performed using a 3.0-Tesla MR system (Discovery MR750, General Electric, Milwaukee, WI, USA), with 8-channel high-resolution radio-frequency head coil. Their ears were packed with earplugs to reduce scanner noise and, tight foam padding was used to minimize head motion as much as possible. The imaging sequences included conventional MR sequence (T2-weighted FLAIR imaging), T1-3D BRAVO sequence and DKI sequence. T2-weighted fast spin-echo sequence was used, with repetition time (TR)/echo time (TE), 9000 ms/124 ms; flip angle (FA), 111°; matrix, 256 × 256; field of view (FOV), 250 × 250 mm; slice thickness, 5 mm without spacing; 20 axial slices. 3D T1-weighted images were obtained with TR/TE, 8.2 ms/3.2 ms; FA, 12°; matrix, 256 × 256; FOV, 256 × 256 mm; slice thickness, 1 mm without spacing; 166 axial slices, covering the entire brain for anatomic reference. Diffusion-weighted images were acquired by a planar imaging (EPI) sequence. The imaging parameters were as follows: TR/TE, 4800 ms/minimum; matrix, 128 × 128; FOV, 240 × 240 mm; voxel size, 1.875 × 1.875 × 3 mm^3^, slice thickness, 3 mm without spacing; 45 axial slices; 15 encoding diffusion directions with two values of *b* (*b* = 1000 and 2000 s/mm^2^) for each direction and 6 nondiffusion-weighted images (*b* = 0 s/mm^2^). All images were visually checked to ensure that only images without visible artifacts and head motion were included in subsequent analyses.

### 2.3. Data Analysis

#### 2.3.1. Data Preprocessing

Data analysis was performed in the Laboratory of Digital Medical Imaging, The First Affiliated Hospital of AUCM. We got the average images of B0 using software of MATLAB R2012a. Then, all diffusion-weighted datasets were preprocessed using the fMRI of the Brain Software Libratory, Version 5.04 (FSL http://fsl.fmrib.ox.ac.uk/fsl/fslwiki/). The row datasets were corrected for eddy current distortion and motion artifacts using affine alignment of each diffusion-weighted image to the null image. After skull stripping to remove background noise and nontissue components, only images with b0 and b1000 s/mm^2^ were employed for DTI fitting because DTI parameters can be only estimated using a monoexponential model based on a single nonzero *b* value [17]. The DTI tensors were calculated by diffusion kurtosis estimator (http://www.nitrc.org/projects/dke) software for whole-brain volumes to produce fractional anisotropy (FA) and mean diffusion (MD). DTI_FA and anatomical images of all subjects were coregistrated and aligned to template of the Montreal Neurological Institute (MNI_152_T1_1mm) space using a nonlinear registration algorithm implemented in FNIRT (FMRIB's nonlinear registration tool). Registrations of DTI_MD parameter and conventional MRI were in the same way.

#### 2.3.2. Intergroup Analysis

Intergroup analysis was performed with *t*-test to investigate variation of microstructure in the brain between the patients of WD and control group. The results of FA parameter and MD parameter intergroup analysis were both corrected with False Discovery Rate (FDR) simulations (*p* = 0.001, *α* = 0.05, cluster size = 326). Thereafter, the activity of copper-dependent oxidase, 24 h urinary excretion of copper, duration of illness, and the modified Goldstein classification were scored and correlated with DTI parameters using ANCOVA correlation analysis.

#### 2.3.3. ROC Curve Analysis

Receiver-operating characteristic (ROC) curves were generated for FA, MD, and MRI signal change ratio, which took the signal of cerebral spinal fluid (CSF) as the reference value for quantitative scoring. The regions of interest (ROIs) for ROC curve analysis were extracted from statistical activation of the intersection of FA and MD maps, including the bilateral HCN and LN. The ROC curve analysis was performed with the SPSS and used to determine the cutoff values associated with optimal sensitivity and specificity for distinguishing WD from the control group.

## 3. Results

### 3.1. Five Subjects of the Patients Were Excluded due to the Failure of Registration

Thus, a total of 30 patients of WD were employed for the final analysis. No significant correlation between DTI parameters and the activity of copper-dependent oxidase, 24 h urinary excretion of copper, and the modified Goldstein classification duration of illness was found.

### 3.2. Results of Intergroup Analysis

**Figure 1:**
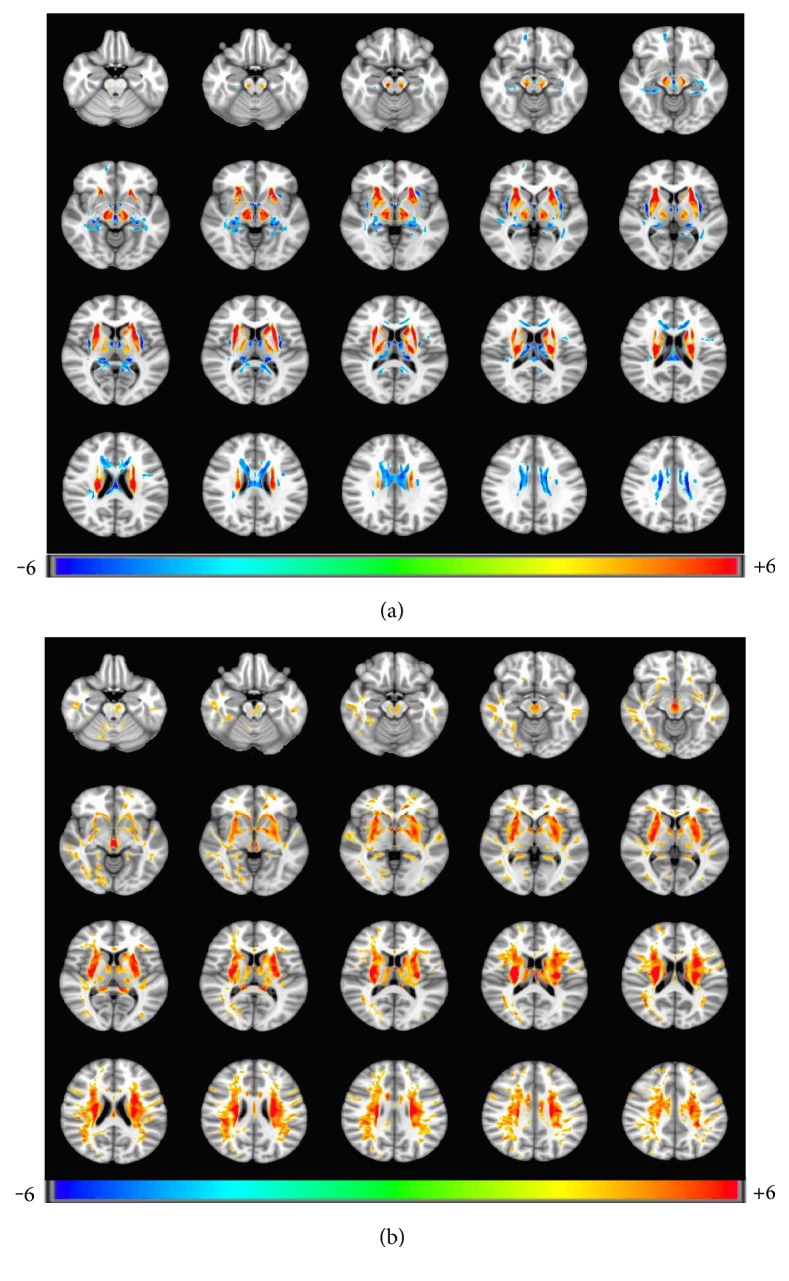
(a) FA parameter differences of brain regions between patients and controls (FDR simulation, (*p* = 0.001, *α* = 0.05, cluster size = 326)). Patients showed increased FA in the bilateral head of the caudate nucleus, lenticular nucleus, ventral thalamus, brain stem, and decreased in mediodorsal thalamus and bilateral extensive matter. (b) MD parameter differences of brain regions between patients and controls (FDR simulation, (*p* = 0.001, *α* = 0.05, cluster size = 326)). Patients showed increased MD in the bilateral head of the caudate nucleus, lenticular nucleus, claustrum, thalamus, brain stem, and extensive white matter.

After being corrected with FDR simulations (*p* = 0.001, *α* = 0.05, cluster size = 326), we found that FA increased in the bilateral head of the caudate nucleus (HCN), lenticular nucleus (LN), ventral thalamus, substantia nigra (SN), red nucleus (RN), right dentate nucleus (DN), and decreased in the mediodorsal thalamus and extensive white matter (Figure 1(a)). MD was increased in HCN, LN, SN, RN, and extensive white matter (Figure 1(b)).

### 3.3. Results of ROC Curve Analysis

**Figure 2:**
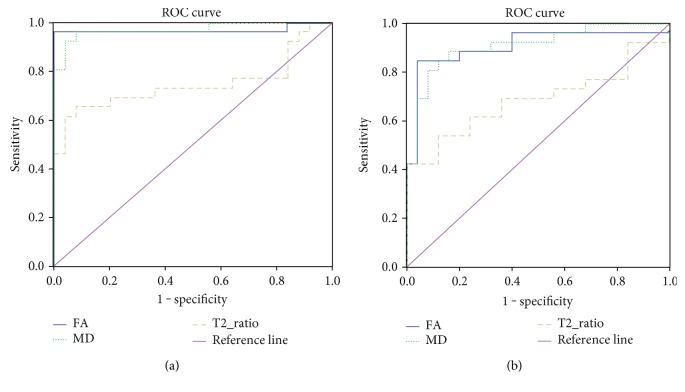
(a) and (b) Graphs show sensitivity and specificity of FA, MD, T2_ratio, and reference standard at bilateral lenticular nucleus. FA and MRI signal change ratio are dimensionless. MD value is given in 1000 square millimeters per second.

The ROC curves with respect to FA, MD, and MRI signal change ratio are shown in Figures 2(a) and 2(b) for the bilateral HCN and LN to discriminate WD from control group, respectively. The cutoff values for FA, MD, and MRI signal change ratio in WD and the corresponding sensitivity and specificity values were shown in Table 2. The areas under ROC curve (AUC) in bilateral LN of FA, MD, and MRI signal change ratio were 0.968, 0.971, and 0.746, respectively, and asymptotic 95% confidence intervals ranged from 0.905 to 1.000 for FA, from 0.925 to 1.000 for MD, and from 0.600 to 0.893 for MRI signal change ratio. The AUC in bilateral HCN of FA, MD and MRI signal change ratio were 0.906, 0.909, and 0.686, respectively, and asymptotic 95% confidence intervals ranged from 0.814 to 0.998 for FA, from 0.827 to 0.991 for MD, and from 0.533 to 0.839 for MRI signal change ratio.

## 4. Discussion

This study applied technique of DTI approach to investigate the microstructural organization of the brain in 35 patients with WD. The results showed diffusion abnormalities in the basal ganglia, thalamus, and brain stem. According to previous reports, it was shown that restricted diffusion in the WD lesions initially followed by facilitated diffusion on the follow-up MRI [18, 19]. These findings were attributed to the presence of cytotoxic edema secondary to inflammation, ischemia secondary to mitochondrial dysfunction, and acute demyelination in the initial phase of disease. In the follow-up imaging, authors attributed this facilitated diffusion to neuronal loss, gliosis, and spongiform changes. Interestingly, in this study, FA increased in the bilateral ventral thalamus but decreased in the bilateral mediodorsal thalamus. Li et al. provided preliminary evidence that the mediodorsal thalamus plays an important role in depression [20]. Previous studies suggested that depression was associated with copper deposition disorder in WD due to central serotonergic deficits. [21, 22] According to our results, there may be a relationship between decreased FA in the mediodorsal thalamus and the presence of depressive symptoms.

These changes of the present study are largely compatible with previous findings in preclinical and clinical WD with various methods. Jadav et al. used the technique of DTI to evaluate white matter abnormalities in WD, finding MD significantly increased in the bilateral internal capsules and midbrain, and FA significantly decreased in the frontal and occipital white matter, bilateral internal capsules, midbrain, and pons, while the images were normal appearing on conventional MRI [15]. Li et al. showed lower FA and higher MD in bilateral thalamus in patients, and Lawrence et al. found higher FA in white matter in the initial phase of the patients [14, 23]. Besides, some other methods were applied to diagnosis and follow-up in WD, either. Susceptibility-weighted imaging was applied by Yang et al. to detect the abnormality in grey matter nuclei. The bilateral head of the caudate nuclei (HCN), globus pallidus (GP), putamen (PUT), thalamus, substantia nigra (SN), and red nucleus (RN) in the WD groups were markedly deposited with paramagnetic mineralization and were significantly different compared to the normal controls, especially the bilateral PUT [24]. Pulai et al. observed that WD patients had reduced N-acetylaspartate/creatine (Cr) and choline (Cho)/Cr ratio in basal ganglia as compared with control subjects in MRS study [25].

WD is an autosomal recessive disorder of copper metabolism due to the dysfunction of a copper-transporting ATP7B, leading to pathological copper accumulation [26]. Cerebral copper deposition mostly localizes to the basal ganglia often inducing rigidity, tremor, dysphagia, and dysarthria [27–29]. However, the mechanism of neurological symptoms caused by pathological copper accumulation in the brain is still not understood. Is there any correlation between the abnormalities in diffusion images and the clinical symptoms? Magnetoencephalographic data of symptomatic patients of WD have been published and demonstrated involvement of a cerebello-thalamo-cortical network in WD tremor generation [6, 30]. Sudmeyer et al. showed strongly significant correlation between unified Parkinson's disease rating scale part III action tremor score and MRI signal change ratio in the head of the caudate nucleus, the globus pallidus, and the substantia nigra in patients of WD [10]. Thus, according to our results, we hypothesized that tremor and dystonia in WD patients might be caused by lesions in cerebello-thalamo-cortical network, which includes the basal ganglia-thalamo-cortical circuits and dentato-rubro-thalamic track.

### 4.1. Basal Ganglia-Thalamo-Cortical Circuits

In the basal ganglia-thalamo-cortical circuits, both the direct pathway (cortico-striato-GPe-STN-Gpi projections) and the indirect pathway (cortico-striato-GPe-STN-Gpi projections) play an important role in mediating voluntary movement [31]. The output nuclei send their axons to motor regions of thalamus, specifically to the ventral anterior and ventrolateral nuclei of the thalamus, which project back to motor regions of the cortex [32, 33]. Lots of previous studies confirmed that medically refractory dystonia and tremor, which are also the most common neurological problems in WD, have been treated with DBS effectively, targeting the Gpi or STN with increased GABA values [31, 34–37]. The increased inhibitory tone of Gpi neurons and the subsequent thalamic inhibition could be the key mechanisms of DBS therapy in dystonia. According to our results, perhaps the motor regions of thalamus were not suppressed effectively due to the lack of GABA receiving from impaired STN and Gpi, or the thalamus failed to accept the neurotransmitter because of lesions in itself, leading to dystonia and tremor finally.

### 4.2. Dentato-Rubro-Thalamic Track

The concept of the dentato-rubro-thalamic (DRT) track, a part of the cerebello-thalamo-cortical network, was first introduced by Coenen et al. [38] The DRT consists mainly of axon fibers arising from the dentate (DN), crossing at the level of the upper pons or inferior colliculus, and entering the contralateral red nucleus (RN), terminating in the ventralis oralis posterior nucleus (VOP) and the ventralis intermedius nucleus (VIM). These nuclei project to the primary motor cortex, concerning with coordination of somatic motor function [39]. Coenen et al. showed the high correlation between the DRT track and tremor with DBS therapy. They used fiber-tracking techniques to visualize and optimize target definition, and the DBS therapy have alleviated tremor effectively. Interestingly, the DRT track connected the target regions [38, 40]. Thus, according to our results, it might be speculated that WD action tremor is caused by lesions in the basal ganglia and thalamus, which disconnect the DRT pathway.

## 5. Conclusion and Limitation

In conclusion, the results of this study reflect altered microstructure and retention of intracellular and extracellular water molecules, which indicates damage in the altered areas. And DTI provides higher sensitivity and specificity than conventional MRI to detect disease of WD. Lesions in the basal ganglia, thalamus, and cerebellum might disconnect the basal ganglia-thalamo-cortical circuits or DRT track and disrupt cerebello-thalamo-cortical network finally, which causes clinical extrapyramidal symptom.

There are several limitations in the present study, which future studies can address. First, deficit of the information of ceruloplasmin levels for controls may decrease the power of our results. Second, in this study, DTI data was collected with only 15 diffusion directions and slice thickness with 3 mm. In the further studies, higher spatial resolution of images will be collected to get more accurate analysis results. Third, our study failed to find any correlation between diffusion parameters and clinical symptoms. Correlation between diffusion metrics and clinical manifestation is important for the establishment of DTI as a clinical biomarker. Future studies may enlarge the sample size to further explore the association between them.

## Figures and Tables

**Table 1:** Details of demographic and clinical features of patients with WD.

At initial presentation	Patients of WD	Controls
Patients (number)	35	30
Males/females (number)	21/14	15/15
Ages (years, mean ± SD)	26.94 ± 6.98	26.2 ± 5.62
Duration of illness (months, mean ± SD)	93.9 ± 60.5	—
Family history	23%	—
KF ring present	100%	—
Extrapyramidal signs	100%	—
Modified Goldstein classification	II~IV	—
24 h urinary excretion of copper (*μ*mmol/L)	873.3 ± 538.8	—
Copper-dependent oxidase activity (L)	0.04 ± 0.06	—

**Table 2:** Statistical values for FA, MD, and MRI signal change ratio in WD.

	Bilateral lenticular nucleus			Bilateral head of caudate nucleus		
	FA	MD	T2_ratio	FA	MD	T2_ratio
Cutoff value	0.158	0.956	0.532	0.160	1.201	0.681
Sensitivity	0.962	0.923	0.615	0.846	0.846	0.423
Specificity	1.00	0.96	0.96	0.96	0.88	1.00
AUC	0.968	0.971	0.746	0.906	0.909	0.686
*p* value	1.02*E*−8	8.13*E*−9	0.03	6.55*E*−7	5.37*E*−7	0.023
Standard error	0.032	0.023	0.075	0.047	0.042	0.078

Note: data are diagnostic sensitivity and specificity values achieved with the best cutoff value, areas under ROC curve in the bilateral lenticular nucleus, and the head of caudate nucleus.
